# Impact of Surgical Margin on the Prognosis of Early Hepatocellular Carcinoma (≤5 cm): A Propensity Score Matching Analysis

**DOI:** 10.3389/fmed.2020.00139

**Published:** 2020-05-07

**Authors:** Han Wang, Hua Yu, You-Wen Qian, Zhen-Ying Cao, Meng-Chao Wu, Wen-Ming Cong

**Affiliations:** ^1^Department of Pathology, Eastern Hepatobiliary Surgery Hospital, The Second Military Medical University, Shanghai, China; ^2^Department of Hepatic Surgery, Eastern Hepatobiliary Surgery Hospital, The Second Military Medical University, Shanghai, China

**Keywords:** hepatocellular carcinoma, early stage, margin, microvascular invasion, liver cirrhosis, recurrence, prognosis

## Abstract

**Aim:** The influence of surgical margin on the prognosis of patients with early solitary hepatocellular carcinoma (HCC) (≤5 cm) is undetermined.

**Methods:** The data of 904 patients with early solitary HCC who underwent liver resection were collected for recurrence-free survival (RFS) and overall survival (OS). Propensity score matching (PSM) was performed to balance the potential bias.

**Results:** Log-rank tests showed that 2 mm was the best cutoff value to discriminate the prognosis of early HCC. Liver resection with a >2 mm surgical margin distance (wide-margin group) led to better 5-year RFS and OS rate compared with liver resection with a ≤2 mm surgical margin distance (narrow-margin group) among patients both before (RFS: 59.1% vs. 39.6%, *P* < 0.001; OS: 85.3% vs. 73.7%, *P* < 0.001) and after PSM (RFS: 56.3% vs. 41.0%, *P* < 0.001; OS: 83.0% vs. 75.0%, *P* = 0.010). Subgroup analysis showed that a wide-margin resection significantly improved the prognosis of patients with microvascular invasion (RFS: *P* < 0.001; OS: *P* = 0.001) and patients without liver cirrhosis (RFS: *P* < 0.001; OS: *P* = 0.001) after PSM. Multivariable Cox regression analysis revealed that narrow-margin resection is associated with poorer RFS [hazard ratio (HR) = 1.781, *P* < 0.001), OS (HR = 1.935, *P* < 0.001], and early recurrence (HR = 1.925, *P* < 0.001).

**Conclusions:** A wide-margin resection resulted in better clinical outcomes than a narrow-margin resection among patients with early solitary HCC, especially for those with microvascular invasion and without cirrhosis. An individual strategy of surgical margin should be formulated preoperation according to both tumor factors and background liver factors.

## Introduction

Hepatocellular carcinoma (HCC) is the most common pathological type of primary liver cancer (1). Solitary HCC up to 5 cm has been authenticated to be low invasive and identified as early HCC with excellent long-term outcomes after curative treatment (2, 3). Although liver resection (LR) of early HCC can result in approximately 75% 5-year overall survival (OS) rate, ~60 to 70% 5-year tumor recurrence rate is still a main clinical concern (4).

Although many optimal strategies for adjuvant therapy after LR, such as transcatheter arterial chemoembolization (TACE) and antivirus treatment, have been confirmed to positively facilitate clinical outcomes, the patients' sufferings, compliance, and finances would prevent the implementation of these measures to some extent (5–7). Therefore, it is still the best way to benefit patients through a one-off radical resection. Concerning both tumor eradication and liver volume preservation, precise hepatectomy is necessary. The parameters reflecting the pathobiological behaviors of tumor and background liver are the best reference for retrospective exploration of strategies for individualized surgical treatment (8).

Microvascular invasion (MVI) is a histological feature that indicates aggressive behavior of HCC (9, 10). The presence of MVI has been regarded as one of the most essential parameters reflecting postoperative recurrence and long-term survival, even in very early-stage HCC (11). A South Korean study showed that solitary HCCs of 2 to 5 cm without MVI could benefit from anatomical resection compared with those with MVI (12). Nevertheless, another study demonstrated anatomical resection was a significantly favorable factor for the MVI positive patients who had single nodule HCC <5 cm (13). Interestingly, a recent study reported that a wide-margin LR improved the long-term prognosis of hepatitis B–related HCC with MVI, which prompted that the low residual rate of tumor cells caused by adequately resection might be the true reason of good prognosis (14).

In addition, liver cirrhosis is one of the most common inducing factors of hepatocarcinogenesis and also directly indicates the feasibility of operation (15, 16). With the development of surgical techniques, some HCC patients with advanced cirrhosis also could achieve curative LR on the premise of perioperative safety (17, 18). However, few studies have explored the impact of surgical margin on the long-term prognosis of those patients. A multicenter study revealed that multiple recurrences near the resection margin or at extrahepatic sites were more frequent in the normal liver HCC patients, whereas solitary recurrence at a distant site was more common in the liver cirrhosis HCC patients (19). Considering opposite significance of different recurrence pattern (20), individual resection range is worthy of discussion in HCC patients with and without liver cirrhosis.

Taking the above results into account, we hypothesize that adequate margin distance may have a positive effect on the prognosis of early HCC patients and therefore carry out the current study. Furthermore, subgroup analysis is performed based on MVI and liver cirrhosis, in order to explore individual therapeutic strategy aiming at both tumor malignancy and background liver function.

## Materials and Methods

### Patients

This study was conducted on patients who underwent LR for primary solitary HCC up to 5 cm at the Eastern Hepatobiliary Surgery Hospital (EHBH) in Shanghai, China, between December 2009 and December 2010. Inclusion criteria included histologically confirmed HCC, solitary tumor up to 5 cm, and Child–Pugh A–B. Exclusion criteria included combined hepatocellular-cholangiocarcinoma, recurrent HCC, macroscopic tumor thrombus in major portal/hepatic veins and bile ducts, extrahepatic metastasis, severe liver dysfunction (such as massive ascites and hepatic encephalopathy), preoperative anticancer treatment, and a history of other malignancy. This study was approved by the Institutional Ethics Committee of the EHBH (no. EHBHKY2015-02-001). Written informed consent was issued by all the patients before operation for using their data for the research.

### Preoperative Assessment and Pathologic Diagnosis

Liver function, tumor markers, complete blood count, blood coagulation function, and hepatitis tests constituted routine preoperative laboratory examinations. Imaging studies included chest x-ray, as well as ultrasonography, contrast-enhanced computed tomography (CT), or magnetic resonance imaging (MRI) of the abdomen. All operations were conducted using a conventional open approach. Intraoperative ultrasonography was used routinely to accurately judge the size, number, location of the lesions, and their relationship to major vascular structures and to rule out additional unknown lesions. Although a wide surgical margin was the aim of the hepatic resection, a grossly negative macroscopic margin without tumor exposure was authorized adequate.

The sampling protocol was implemented by pathologists based on the 7-point baseline sampling protocol as previously reported (21). Three experienced pathologists evaluated all sections independently. The tumor size was on account of the largest dimension of the tumor in the resection specimen. Microvascular invasion was defined as tumors within a vascular space lined by endothelium that was visible only on microscopy (22). Liver cirrhosis was defined as at least one pseudolobule was seen in the liver tissue microscopically. The Edmondson–Steiner classification was used to determine HCC differentiation (23). The definition of anatomical resection was based on the Brisbane 2000 Nomenclature of Liver Anatomy and Resections, and non-anatomical resection indicated wedge/limited resection (24). Anatomical resection was defined as the systematic removal of a hepatic territory confined by tumor-bearing portal branches, whereas non-anatomical resection was defined as local resection or enucleation regardless of Couinaud segments.

### Follow-Up

All patients were followed up once every 2 months in the first year and once every 3 months thereafter. Follow-up investigations consisted of ultrasonographic scans, CT, or MRI with serum α-fetoprotein (AFP). The recurrence-free survival (RFS) was defined as the time from initial treatment to tumor recurrence or censored. Overall survival was defined as the interval between treatment and death or the date of the last follow-up visit. Follow-up data were censored until 60 months.

Relapse was considered as suspicious imaging findings or a biopsy-confirmed tumor. As the diagnosis of tumor recurrence was certain, the therapeutic options were decided according to number of tumors, tumor site, liver function, and general patient condition. Treatment methods included surgical re-resection, ablation, TACE, and other selections, such as liver transplantation, chemotherapy, percutaneous ethanol injection therapy, radiotherapy, sorafenib, and translational Chinese medicine.

### X-Tile and Best Cut-Off Value of Surgical Margin

In the previous studies, macroscopic no margin (25), 5 mm (26), and 1 cm (14) all have been employed as the cutoff values of surgical margin. However, these values were based more on the clinical experience, rather than evidence. Considering the high perioperative safety of early HCC, prognosis is regarded as the best reference to explore the cutoff value of surgical margin distance. In our study, X-tile plots were used for assessment of surgical margin, which was represented as distance value and optimization of cut-point based on RFS and OS (27). Statistical significance was assessed using the cutoff score derived from 904 HCC cases by a standard log-rank method, with *P*-values obtained from a lookup table. The distance value that obtained the biggest χ^2^ value was employed as the cutoff point that discriminated “wide-margin” and “narrow-margin” LR.

### Statistical Analysis

Continuous variables were described as mean ± standard deviation. Categorical variables were presented as frequencies (percentages). Statistical calculation of categorical and continuous variables was performed using the χ^2^ test or the Fisher exact test and the Student *t*-test or the Mann-Whitney *U*-test, when appropriate. Survival analyses were conducted utilizing the Kaplan–Meier method, the log-rank test. The Cox proportional hazards model was used in exploring independent prognostic factors of RFS and OS. The predictive factors of MVI were determined using univariable and multivariable logistic regression models. Variables with *P* < 0.1 in univariable analysis of the Cox regression and logistic regression were selected for screening of the multivariable model.

The influence of confounding factors and selection bias were reduced by propensity score matching (PSM) (28). All variables with potential differences (*P* < 0.2) were entered into the PSM model. The matching variables included age, albumin (ALB), γ-glutamyl transpeptidase (GGT), alkaline phosphatase (ALP), platelets, prothrombin time (PT), hepatitis B virus deoxyribonucleic acid (HBV DNA) load, Child–Pugh classification, hepatectomy method, transfusion, diameter, cirrhosis, capsule, and MVI. Considering the high correlation between PT and international normalized ratio, we selected PT in propensity matching. The logistic regression analysis was performed using the nearest neighbor matching to estimate the propensity score. The ratio for matching was established at 1:1 using a caliper of width equal to 0.05 of the standard deviation of the logit of the propensity score. No discards or replacements were employed. All *P*-values were 2-tailed, with *P* < 0.05 considered statistically significant. All statistical analyses were conducted with the software package SPSS 24.0 (IBM, New York, USA).

## Results

### Suitable Cut-Points of Surgical Margin Distance

**Figure 1:**
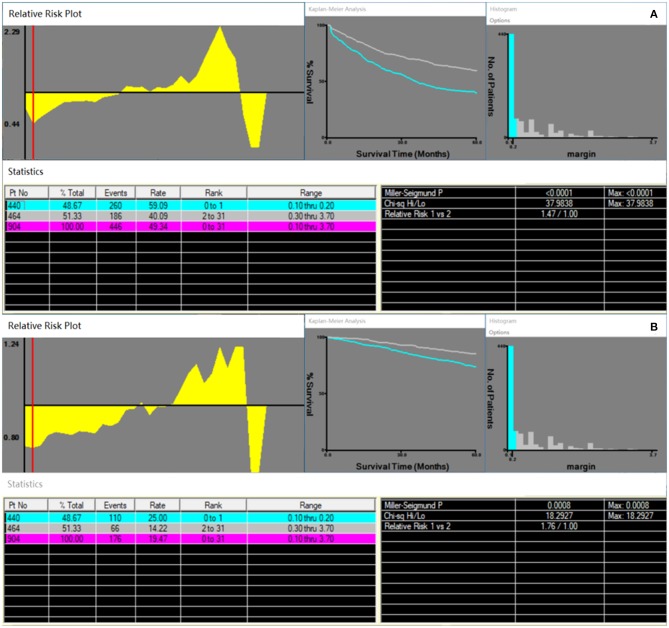
Prognosis analyses by X-tile plot based on the distance of surgical margin. X-tile plots showing **(A)** recurrence-free survival; **(B)** overall survival.

In this study, 904 patients who met the inclusion criteria were included. Based on the X-tile plots results, both RFS and OS of 904 HCC patients obtained the biggest discrimination when using 2 mm as the cutoff value of surgical margin distance (Figure 1A: RFS, *P* < 0.0001, χ^2^ = 37.9838; Figure 1B: OS, *P* = 0.0008, χ^2^ = 18.2927). As such, patients who underwent a ≤2 mm surgical margin LR were defined as “narrow-margin” group (*n* = 440); otherwise, “wide-margin” group (*n* = 464). Supplementary Table 1 shows the χ^2^ and *P*-values when other surgical margin distance values were used as the cutoff point.

### Patient Demographics

Table 1 shows the baseline characteristics of all the patients. After PSM analysis, 335 pairs of patients were selected, and comparisons of all parameters between the two groups revealed no significant differences (Table 1, all *P* > 0.05). In the whole patients, perioperative mortality (≤60 days) occurred in 5 patients (0.55%), of which 4 and 1 patients underwent narrow- and wide-margin LR.

**Table 1:** Baseline clinicopathological characteristics of patients.

**Variables**	**Before PSM**			**After PSM**		
	**Wide margin (*n* = 464)**	**Narrow margin (*n =* 440)**	***P*-value**	**Wide margin (*n =* 335)**	**Narrow margin (*n =* 335)**	***P*-value**
Sex			0.655			0.831
Male	389 (83.8%)	364 (82.7%)		284 (84.8%)	282 (84.2%)	
Female	75 (16.2%)	76 (17.3%)		51 (15.2%)	53 (15.8%)	
Age, year	51.37 ± 9.67	52.99 ± 10.08	0.016	51.98 ± 9.60	51.82 ± 10.06	0.897
TBIL, μmol/L	14.63 ± 5.76	14.83 ± 7.10	0.995	14.61 ± 5.81	14.40 ± 5.23	0.571
TP, g/L	73.96 ± 5.49	73.64 ± 5.76	0.388	73.88 ± 5.40	74.09 ± 5.76	0.782
ALB, g/L	42.91 ± 3.61	42.31 ± 4.06	0.020	42.59 ± 3.65	43.01 ± 3.76	0.150
ALT, U/L	41.05 ± 27.37	40.05 ± 29.16	0.770	42.61 ± 29.42	39.66 ± 30.66	0.318
AST, U/L	33.38 ± 17.24	34.96 ± 19.75	0.203	34.60 ± 18.52	33.44 ± 19.37	0.349
GGT, U/L	59.56 ± 60.84	76.15 ± 86.05	<0.001	66.08 ± 67.67	62.95 ± 57.52	0.903
ALP, U/L	76.45 ± 22.08	81.94 ± 26.02	0.002	78.63 ± 22.46	77.71 ± 22.82	0.624
AFP, ng/mL	272.08 ± 429.36	276.17 ± 437.56	0.600	294.76 ± 442.88	288.75 ± 445.01	0.302
CA199, ng/mL	25.19 ± 23.13	23.42 ± 20.27	0.637	26.60 ± 25.06	21.78 ± 18.22	0.072
WBC, ×10^9^/L	5.08 ± 1.61	5.03 ± 1.70	0.395	4.98 ± 1.63	5.22 ± 1.62	0.110
RBC, ×10^9^/L	4.66 ± 0.50	4.64 ± 0.52	0.236	4.64 ± 0.51	4.71 ± 0.49	0.390
PLT, ×10^9^/L	147.44 ± 58.44	137.88 ± 58.30	0.016	143.74 ± 58.54	145.52 ± 55.21	0.619
INR	1.00 ± 0.08	1.01 ± 0.09	0.143	1.01 ± 0.08	1.00 ± 0.08	0.734
PT, s	12.03 ± 0.93	12.16 ± 1.06	0.135	12.08 ± 1.00	12.05 ± 0.98	0.725
HBsAg			0.294			0.347
Positive	424 (91.4%)	393 (89.3%)		301 (89.9%)	308 (91.9%)	
Negative	40 (8.6%)	47 (10.7%)		34 (10.1%)	27 (8.1%)	
HBsAb			0.728			0.360
Positive	67 (14.4%)	60 (13.6%)		287 (85.7%)	295 (88.1%)	
Negative	397 (85.6%)	380 (86.4%)		48 (14.3%)	40 (11.9%)	
HBeAg			0.623			0.933
Positive	143 (30.8%)	129 (29.3%)		101 (30.1%)	100 (29.9%)	
Negative	321 (69.2%)	311 (70.7%)		234 (69.9%)	235 (70.1%)	
HBeAb			0.703			0.529
Positive	345 (74.4%)	332 (75.5%)		250 (74.6%)	257 (76.7%)	
Negative	119 (25.6%)	108 (24.5%)		85 (25.4%)	78 (23.3%)	
HBcAb			1.000			0.682
Positive	460 (99.1%)	437 (99.3%)		331 (98.8%)	333 (99.4%)	
Negative	4 (0.9%)	3 (0.7%)		4 (1.2%)	2 (0.6%)	
HBV DNA load			0.164			0.643
≤10^3^ IU/mL	220 (47.4%)	229 (52.0%)		171 (51.0%)	165 (49.3%)	
>10^3^ IU/mL	244 (52.6%)	211 (48.0%)		164 (49.0%)	170 (50.7%)	
Child–Pugh			0.001			1.000
A	461 (99.4%)	423 (96.1%)		332 (99.1%)	331 (98.8%)	
B	3 (0.6%)	17 (3.9%)		3 (0.9%)	4 (1.2%)	
Hepatectomy			0.150			0.460
Anatomical	77 (16.6%)	58 (13.2%)		57 (17.0%)	50 (14.9%)	
Non-anatomical	387 (83.4%)	382 (86.8%)		278 (83.0%)	285 (85.1%)	
Transfusion			<0.001			0.430
Yes	30 (6.5%)	79 (18.0%)		29 (8.7%)	35 (10.4%)	
No	434 (93.5%)	361 (82.0%)		306 (91.3%)	300 (89.6%)	
Pringle maneuver			0.483			0.430
Yes	392 (84.5%)	379 (86.1%)		286 (85.4%)	293 (87.5%)	
No	72 (15.5%)	61 (13.9%)		49 (14.6%)	42 (12.5%)	
Diameter, cm	2.94 ± 1.00	3.20 ± 1.05	<0.001	3.12 ± 1.01	3.10 ± 1.03	0.812
Cirrhosis			0.001			0.632
Yes	263 (56.7%)	298 (67.7%)		213 (63.6%)	207 (61.8%)	
No	201 (43.3%)	142 (32.3%)		122 (36.4%)	128 (38.2%)	
Capsule			0.197			0.733
Yes	391 (84.3%)	384 (87.3%)		289 (86.3%)	292 (87.2%)	
No	73 (15.7%)	56 (12.7%)		46 (13.7%)	43 (12.8%)	
MVI			0.003			0.636
Yes	199 (42.9%)	147 (33.4%)		136 (40.6%)	130 (38.8%)	
No	265 (57.1%)	293 (66.6%)		199 (59.4%)	205 (61.2%)	
ES grade			0.360			0.806
I–II	147 (31.7%)	152 (34.5%)		113 (33.7%)	110 (32.8%)	
III–IV	317 (68.3%)	288 (65.5%)		222 (66.3%)	225 (67.2%)	

*PSM, propensity score matching; MVI, microvascular invasion; TBIL, total bilirubin; TP, total protein; ALB, albumin; ALT, alanine transaminase; AST, aspartate aminotransferase; GGT, γ-glutamyl transpeptidase; ALP, alkaline phosphatase; AFP, α fetal protein; CA199, carbohydrate antigen 19-9; WBC, white blood cells; RBC, red blood cells; PLT, platelets; INR, international normalized ratio; PT, prothrombin time; HBsAg, hepatitis B surface antigen; HBsAb, hepatitis B surface antibody; HBeAg, hepatitis B e antigen; HBeAb, hepatitis B e antibody; HBcAb, hepatitis B c antibody; HBV DNA, hepatitis B virus deoxyribonucleic acid; MVI, microvascular invasion; ES, Edmondson–Steiner*.

### Impact of Surgical Margin on Prognosis

**Figure 2:**
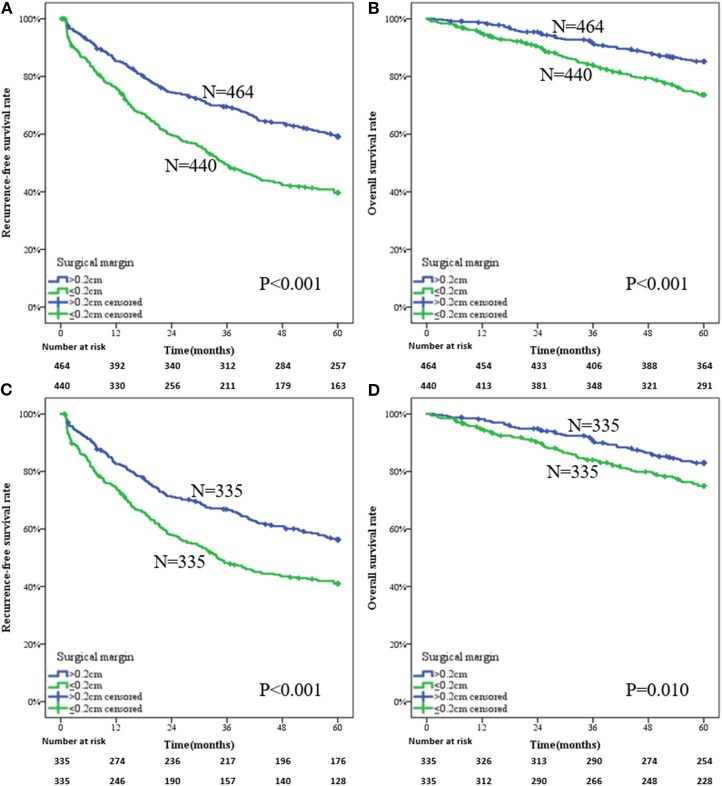
Survival analysis of a wide-margin vs. a narrow-margin liver resection for the early solitary hepatocellular carcinoma. **(A)** Recurrence-free survival in the whole patients, **(B)** overall survival in the whole patients, **(C)** recurrence-free survival in the patients after propensity score matching, **(D)** overall survival in the patients after propensity score matching.

For the whole patients, a narrow-margin LR provided a more adverse prognosis than a wide-margin LR, with 1-, 3-, and 5-year RFS rates being 76.1, 49.3, and 39.6% vs. 85.3, 69.5, and 59.1%, respectively (Figure 2A, *P* < 0.001). The mean RFSs in narrow- and wide-margin groups were 35.32 and 44.67 months, respectively. The corresponding OS rates were 95.2, 84.1, and 73.7% vs. 98.7, 91.2, and 85.3%, respectively (Figure 2B, *P* < 0.001). The mean OS in narrow- and wide-margin groups were 52.16 and 55.88 months, respectively.

For the patients of the PSM group, the results also showed that narrow-margin LR indicated a poorer prognosis compared with wide-margin LR. The 1-, 3-, and 5-year RFS rates in narrow- and wide-margin groups were 74.4, 48.2, and 41.0% vs. 82.6, 66.9, and 56.3%, respectively (Figure 2C, *P* < 0.001). The mean time of RFS was 35.00 months in the narrow-margin group and 43.09 months in the wide-margin group. The 1-, 3-, and 5-year OS rates in corresponding groups were 94.9, 84.0, and 75.0% vs. 98.2, 90.3, and 83.0%, respectively (Figure 2D, *P* = 0.010). The mean time of OS was 52.26 months in the narrow-margin group and 55.33 months in the wide-margin group.

### Subgroup Survival Analysis Based on MVI and Liver Cirrhosis

**Figure 3:**
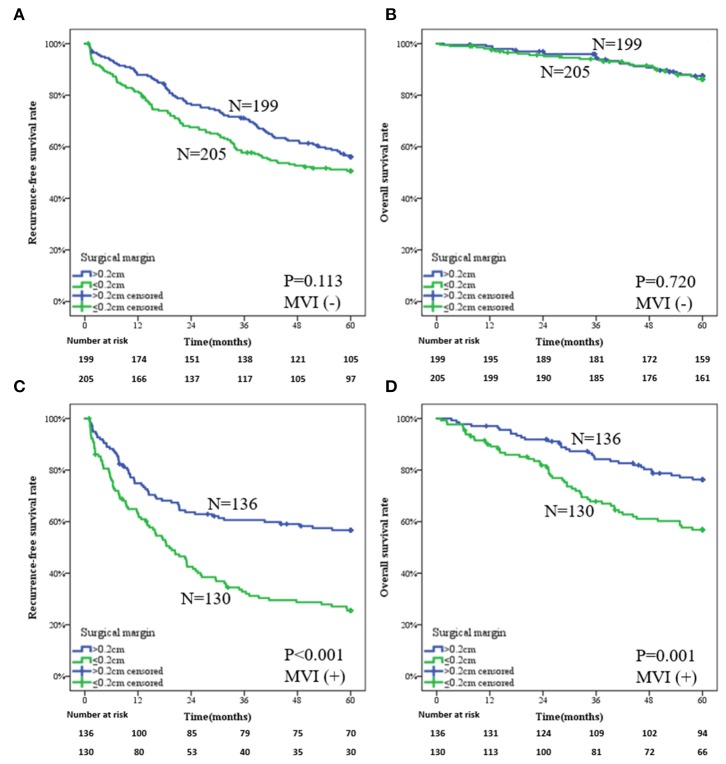
Subgroup analysis of a wide-margin vs. a narrow-margin liver resection for the patients in the propensity score matching group. **(A)** Recurrence-free survival in the patients without microvascular invasion, **(B)** overall survival in the patients without microvascular invasion, **(C)** recurrence-free survival in the patients with microvascular invasion, **(D)** overall survival in the patients with microvascular invasion.

Subgroup analysis for the whole patients showed that wide-margin surgery benefits more on prognosis for both MVI-negative and MVI-positive patients (Supplementary Figure 1). However, for the patients in the PSM group, the influence of surgical margin on prognosis was distinguished by presence of MVI. For the patients without MVI, both RFS and OS showed no significant difference in narrow- and wide-margin groups. The 1-, 3-, and 5-year RFS rates in the two groups were 81.4, 57.7, and 50.6% vs. 87.9, 71.1, and 56.1%, respectively (Figure 3A, *P* = 0.113). The 1-, 3-, and 5-year OS rates in the two groups were 98.0, 94.0, and 86.2% vs. 99.0, 94.4, and 87.5%, respectively (Figure 3B, *P* = 0.720). For the patients with MVI, the narrow-margin group obtained a higher recurrence rate than did the wide-margin group. The 1-, 3-, and 5-year RFS rates in the two groups were 63.3, 32.8, and 25.4% vs. 74.9, 60.6, and 56.7%, respectively (Figure 3C, *P* < 0.001). The 1-, 3-, and 5-year OS rates in the two groups were 89.9, 67.8, and 56.8% vs. 97.1, 84.2, and 76.3%, respectively (Figure 3D, *P* = 0.001). The predictive model of MVI is shown in Supplementary Table 2.

**Figure 4:**
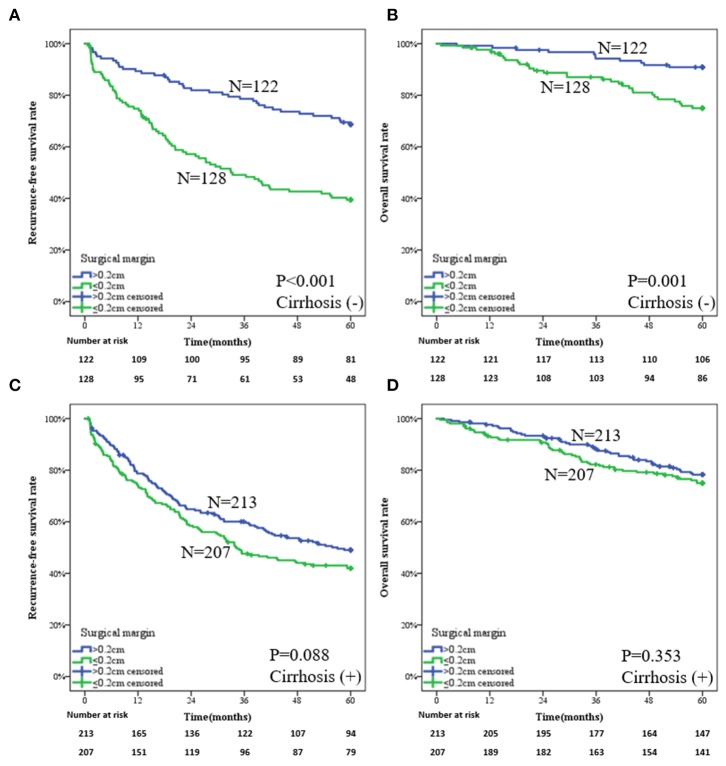
Subgroup analysis of a wide-margin vs. a narrow-margin liver resection for the patients in the propensity score matching group. **(A)** Recurrence-free survival in the patients without liver cirrhosis, **(B)** overall survival in the patients without liver cirrhosis, **(C)** recurrence-free survival in the patients with liver cirrhosis, **(D)** overall survival in the patients with liver cirrhosis.

Subgroup analysis for the whole patients based on the liver cirrhosis is shown in Supplementary Figure 2. The impact of surgical margin on prognosis was distinguished by presence of liver cirrhosis for the patients of the PSM group. For the patients without liver cirrhosis, those who underwent a wide-margin LR obtained better clinical outcomes than did patients who underwent a narrow-margin LR. The 1-, 3-, and 5-year RFS rates in the two groups were 89.3, 78.6, and 68.7% vs. 74.8, 49.1, and 39.5%, respectively (Figure 4A, *P* < 0.001). The 1-, 3-, and 5-year OS rates in the two groups were 99.2, 94.2, and 90.9% vs. 97.6, 87.0, and 75.0%, respectively (Figure 4B, *P* = 0.001). For the patients with liver cirrhosis, there is no statistical difference by wide- or narrow-margin LR. The 1-, 3-, and 5-year RFS rates in the two groups were 78.7, 60.1, and 49.0% vs. 74.2, 47.6, and 42.0%, respectively (Figure 4C, *P* = 0.088). The 1-, 3-, and 5-year OS rates in the two groups were 97.6, 88.0, and 78.2% vs. 93.2, 82.2, and 75.0%, respectively (Figure 4D, *P* = 0.353).

### Prognostic Factors of RFS and OS

Results on the Cox regression analysis of the whole patients for RFS and OS are revealed in Table 2. Multivariable analysis included male, lower ALB, higher aspartate aminotransferase (AST), higher GGT, higher PT, presence of MVI, and narrow-margin LR as the independent prognostic determinants of poorer RFS. Higher GGT, larger tumor diameter, liver cirrhosis, presence of MVI, and narrow-margin LR were independently correlated with poorer OS.

**Table 2:** Prognosis factors of recurrence-free survival and overall survival in the whole patients.

**Variables**	**Recurrence-free survival**				**Overall survival**			
	**Univariable**		**Multivariable**		**Univariable**		**Multivariable**	
	**Hazard ratio (95% CI)**	***P*-value**	**Hazard ratio (95% CI)**	***P*-value**	**Hazard ratio (95%CI)**	***P*-value**	**Hazard ratio (95% CI)**	***P*-value**
Sex, male	1.487 (1.135, 1.948)	0.004	1.696 (1.285, 2.238)	<0.001	1.171 (0.777, 1.765)	0.450		
Age, year	1.000 (0.990, 1.009)	0.941			1.020 (1.005, 1.035)	0.010		
TBIL, μmol/L	1.009 (0.995, 1.023)	0.197			1.014 (0.991, 1.037)	0.231		
TP, g/L	0.968 (0.951, 0.984)	<0.001			0.973 (0.947, 0.999)	0.043		
ALB, g/L	0.926 (0.903, 0.949)	<0.001	0.955 (0.929, 0.982)	0.001	0.897 (0.862, 0.932)	<0.001		
ALT, U/L	1.005 (1.003, 1.008)	<0.001			1.001 (0.995, 1.006)	0.852		
AST, U/L	1.012 (1.007, 1.016)	<0.001	1.006 (1.002, 1.011)	0.010	1.009 (1.002, 1.016)	0.008		
GGT, U/L	1.003 (1.002, 1.004)	<0.001	1.001 (1.000, 1.002)	0.042	1.003 (1.001, 1.004)	<0.001	1.002 (1.000, 1.003)	0.026
ALP, U/L	1.006 (1.002, 1.010)	0.002			1.011 (1.006, 1.017)	<0.001		
AFP, ng/mL	1.000 (1.000, 1.000)	0.285			1.000 (1.000, 1.001)	0.149		
CA199, ng/mL	1.004 (1.000, 1.008)	0.036			1.005 (0.999, 1.011)	0.107		
WBC, ×10^9^/L	0.932 (0.879, 0.988)	0.019			0.965 (0.879, 1.060)	0.456		
RBC, ×10^9^/L	0.896 (0.744, 1.081)	0.252			0.577 (0.435, 0.765)	<0.001		
PLT, ×10^9^/L	0.997 (0.995, 0.998)	<0.001			0.997 (0.994, 0.999)	0.017		
INR	33.181 (11.210, 98.218)	<0.001			28.473 (5.268, 153.890)	<0.001		
PT, s	1.339 (1.224, 1.466)	<0.001	1.181 (1.069, 1.304)	0.001	1.326 (1.152, 1.525)	<0.001		
HBsAg, positive	1.149 (0.831, 1.590)	0.401			0.638 (0.414, 0.981)	0.040		
HBsAb, positive	0.928 (0.707, 1.218)	0.590			1.113 (0.739, 1.677)	0.609		
HBeAg, positive	1.261 (1.036, 1.534)	0.020			1.220 (0.893, 1.666)	0.211		
HBeAb, positive	0.939 (0.761, 1.159)	0.560			0.818 (0.589, 1.134)	0.228		
HBcAb, positive	1.886 (0.470, 7.565)	0.371			0.444 (0.142, 1.390)	0.163		
HBV DNA load, >10^3^ IU/mL	1.336 (1.108, 1.610)	0.002			1.192 (0.886, 1.603)	0.246		
Child–Pugh, B	1.912 (1.122, 3.257)	0.017			2.042 (0.904, 4.611)	0.086		
Hepatectomy, anatomical	0.814 (0.620, 1.068)	0.138			0.758 (0.480, 1.195)	0.233		
Transfusion, yes	1.233 (0.938, 1.622)	0.134			1.306 (0.855, 1.994)	0.217		
Pringle maneuver, yes	0.996 (0.767, 1.294)	0.977			1.012 (0.668, 1.535)	0.954		
Diameter, cm	1.101 (1.006, 1.204)	0.036			1.304 (1.128, 1.506)	<0.001	1.279 (1.103, 1.483)	0.001
Cirrhosis, yes	1.511 (1.237, 1.845)	<0.001			1.727 (1.239, 2.407)	<0.001	1.529 (1.085, 2.155)	0.015
Capsule, no	1.161 (0.894, 1.507)	0.264			1.529 (1.052, 2.223)	0.026		
MVI, yes	1.506 (1.249, 1.816)	<0.001	1.667 (1.378, 2.017)	<0.001	2.369 (1.759, 3.192)	<0.001	2.578 (1.905, 3.488)	<0.001
ES grade, III–IV	1.046 (0.859, 1.274)	0.654			1.754 (1.237, 2.489)	0.002		
Surgical margin, narrow	1.795 (1.487, 2.168)	<0.001	1.781 (1.469, 2.160)	<0.001	1.924 (1.418, 2.611)	<0.001	1.935 (1.413, 2.650)	<0.001

*TBIL, total bilirubin; TP, total protein; ALB, albumin; ALT, alanine transaminase; AST, aspartate aminotransferase; GGT, γ-glutamyl transpeptidase; ALP, alkaline phosphatase; AFP, α fetal protein; CA199, carbohydrate antigen 19-9; WBC, white blood cells; RBC, red blood cells; PLT, platelets; INR, international normalized ratio; PT, prothrombin time; HBsAg, hepatitis B surface antigen; HBsAb, hepatitis B surface antibody; HBeAg, hepatitis B e antigen; HBeAb, hepatitis B e antibody; HBcAb, hepatitis B c antibody; HBV DNA, hepatitis B virus deoxyribonucleic acid; MVI, microvascular invasion; ES, Edmondson–Steiner*.

### Prognostic Factors of Early and Late Tumor Recurrence

The independent risk factors for early tumor recurrence (<2 years) were assessed among all the 904 patients, whereas the factors associated with late recurrence were analyzed among the 596 patients who had a postoperative recurrence after 2 years or more or did not recur (Table 3). The multivariable analyses indicated that the following factors were related to early relapse: AST, AFP, HBV DNA load, tumor capsule, MVI, and surgical margin. Simultaneously, late relapse was associated with GGT, Child–Pugh classification, and liver cirrhosis. The recurrence pattern of all patients is shown in Supplementary Table 3.

**Table 3:** Prognosis factors of early and late recurrence in the whole patients.

**Variables**	**Early recurrence-free survival**				**Late recurrence-free survival**			
	**Univariable**		**Multivariable**		**Univariable**		**Multivariable**	
	**Hazard ratio (95% CI)**	***P*-value**	**Hazard ratio (95% CI)**	***P*-value**	**Hazard ratio (95% CI)**	***P*-value**	**Hazard ratio (95% CI)**	***P*-value**
Sex, male	1.730 (1.210, 2.474)	0.003			1.177 (0.777, 1.783)	0.443		
Age, year	0.994 (0.983, 1.005)	0.299			1.011 (0.995, 1.027)	0.194		
TBIL, μmol/L	1.010 (0.994, 1.026)	0.211			1.006 (0.979, 1.035)	0.661		
TP, g/L	0.955 (0.935, 0.976)	<0.001			0.991 (0.963, 1.020)	0.541		
ALB, g/L	0.919 (0.891, 0.947)	<0.001			0.940 (0.900, 0.982)	0.005		
ALT, U/L	1.006 (1.003, 1.009)	<0.001			1.004 (0.999, 1.009)	0.098		
AST, U/L	1.011 (1.006, 1.016)	<0.001	1.008 (1.003, 1.013)	0.002	1.013 (1.006, 1.021)	<0.001		
GGT, U/L	1.003 (1.002, 1.004)	<0.001			1.003 (1.001, 1.005)	0.003	1.003 (1.001, 1.005)	0.014
ALP, U/L	1.006 (1.001, 1.010)	0.012			1.006 (1.000, 1.013)	0.054		
AFP, ng/mL	1.001 (1.000, 1.001)	<0.001	1.000 (1.000, 1.001)	0.002	0.999 (0.998, 0.999)	<0.001		
CA199, ng/mL	1.004 (0.999, 1.008)	0.162			1.006 (0.999, 1.013)	0.096		
WBC, ×10^9^/L	0.939 (0.873, 1.009)	0.087			0.918 (0.830, 1.016)	0.100		
RBC, ×10^9^/L	0.934 (0.743, 1.175)	0.561			0.824 (0.596, 1.140)	0.242		
PLT, ×10^9^/L	0.998 (0.996, 1.000)	0.057			0.994 (0.991, 0.996)	<0.001		
INR	40.520 (10.818, 151.766)	<0.001			22.048 (3.270, 148.652)	0.001		
PT, s	1.362 (1.221, 1.520)	<0.001			1.293 (1.104, 1.515)	0.001		
HBsAg, positive	1.262 (0.831, 1.917)	0.274			0.980 (0.584, 1.644)	0.937		
HBsAb, positive	0.776 (0.540, 1.115)	0.171			1.222 (0.806, 1.851)	0.345		
HBeAg, positive	1.152 (0.902, 1.471)	0.258			1.494 (1.076, 2.075)	0.017		
HBeAb, positive	1.071 (0.819, 1.399)	0.617			0.744 (0.528, 1.048)	0.091		
HBcAb, positive	2.480 (0.348, 17.668)	0.364			1.292 (0.181, 9.233)	0.798		
HBV DNA load, >10^3^ IU/mL	1.440 (1.142, 1.817)	0.002	1.328 (1.045, 1.687)	0.021	1.161 (0.846, 1.592)	0.355		
Child–Pugh, B	1.497 (0.741, 3.024)	0.260			3.015 (1.332, 6.829)	0.008	2.622 (1.151, 5.971)	0.022
Hepatectomy, anatomical	0.878 (0.631, 1.221)	0.438			0.703 (0.435, 1.136)	0.150		
Transfusion, yes	1.273 (0.915, 1.771)	0.152			1.153 (0.705, 1.885)	0.571		
Pringle maneuver, yes	0.821 (0.607, 1.111)	0.201			1.590 (0.933, 2.708)	0.088		
Diameter, cm	1.213 (1.085, 1.356)	0.001			0.920 (0.790, 1.071)	0.282		
Cirrhosis, yes	1.439 (1.124, 1.844)	0.004			1.650 (1.175, 2.317)	0.004	1.518 (1.075, 2.145)	0.018
Capsule, no	1.417 (1.048, 1.917)	0.024	1.361 (1.000, 1.852)	0.050	0.718 (0.421, 1.223)	0.223		
MVI, yes	1.916 (1.523, 2.411)	<0.001	1.923 (1.520, 2.433)	<0.001	0.915 (0.648, 1.292)	0.613		
ES grade, III–IV	1.196 (0.931, 1.536)	0.161			0.825 (0.597, 1.140)	0.244		
Surgical margin, narrow	1.786 (1.413, 2.258)	<0.001	1.925 (1.519, 2.440)	<0.001	1.812 (1.319, 2.490)	<0.001		

*TBIL, total bilirubin; TP, total protein; ALB, albumin; ALT, alanine transaminase; AST, aspartate aminotransferase; GGT, γ-glutamyl transpeptidase; ALP, alkaline phosphatase; AFP, α fetal protein; CA199, carbohydrate antigen 19-9; WBC, white blood cells; RBC, red blood cells; PLT, platelets; INR, international normalized ratio; PT, prothrombin time; HBsAg, hepatitis B surface antigen; HBsAb, hepatitis B surface antibody; HBeAg, hepatitis B e antigen; HBeAb, hepatitis B e antibody; HBcAb, hepatitis B c antibody; HBV DNA, hepatitis B virus deoxyribonucleic acid; MVI, microvascular invasion; ES, Edmondson–Steiner*.

## Discussion

Hepatic resection has been accepted as the preferred treatment method for single-nodule HCC patients with well-preserved liver function (29). Nevertheless, it is still worth exploring how to cut the tumors thoroughly and preserve liver volume to the greatest extent. However, there is still controversial on patient selection, standard of surgery, and its distributed impact on prognosis (30, 31). Given that large-size tumor calls for more stringent method about volume of residual liver parenchyma, and multinodular HCC is difficult to make statistical assessment, solitary early HCCs up to 5 cm served as the research population of this study. For the best treatment method of those HCCs, LR and ablation are always employed for comparison. A real-world study showed that LR possessed superior intrahepatic control rate than radiofrequency ablation in most conditions of HCC smaller than 5 cm (32). Another study based on the Surveillance, Epidemiology, and End Results database also revealed that LR may confer more survival benefits than radiofrequency ablation for different tumor sizes measuring up to 5 cm and may be an appropriate first-line treatment (33). For the recurrent early HCC, a randomized clinical trial suggested that repeat hepatectomy was associated with better OS than radiofrequency ablation among patients with a tumor diameter from 3 to 5 cm. Therefore, LR still may have advantages in the radical removal of tumor. Furthermore, most reports indicated that the benefit of a wide margin of LR is more prominent (34–36). Therefore, we explore the influence of surgical margin on the prognosis of early HCC patients.

Taking 2 mm as the cutoff point of surgical margin, the results suggested that the surgical extent of LR significantly affects disease recurrence and long-term survival in treating early HCC, and we observed a significant 19.5% decrease in the 5-year recurrence rate and an 11.6% increase in the 5-year OS rate for the wide-margin LR group when compared to the narrow-margin LR group, and similar results were demonstrated after PSM. In addition, a low perioperative mortality could be maintained. All these results prompted that a wide-margin LR is an effective and safety intervention.

Previous viewpoint reminded that the necessity of adequate surgical margin was mainly to completely remove MVI and its subsequent portal dissemination and satellitosis (37). Therefore, subgroup analyses were performed to explore the impact of MVI on surgical margin. For the whole patients, survival analysis showed that patients with or without MVI all could benefit from a wide-margin surgery. We deemed it might be attributed to the imbalance of baseline characteristics, and therefore we performed a PSM analysis. It was remarkable that our study revealed that wide-margin LR only significantly improved the prognosis of early HCC patients with MVI instead of those without MVI after PSM. Our results also corroborated that MVI was significantly associated with early recurrence rather than late recurrence. As such, integrating the close relationship between early recurrence and intrahepatic relapse of residual tumor, a wide-margin LR is a useful intervention of MVI-positive early HCC patients. In addition, liver cirrhosis represents the baseline liver state of patients and is associated with recurrence (38). In our study, the whole patients who were with absence of liver cirrhosis could benefit from a wide-margin surgery in RFS and OS, and those who were with presence of liver cirrhosis could not benefit from a wide-margin surgery in OS, whereas after PSM, it was remarkable that our results suggest that only patients without liver cirrhosis could apparently benefit from a wide-margin LR. We suppose that liver fibrosis is the inducement of HCC and simultaneously the mechanism to repair chronic injury. Therefore, a certain degree of proliferation of fibrous tissue in liver can limit the occurrence of tumor micrometastasis, especially the occurrence of MVI. Our results also showed that presence of tumor capsule, which reflects hepatic fibrosis response, was obviously associated with low risk of MVI and early recurrence. Therefore, a detailed assessment of MVI and liver cirrhosis before operation is essential. We also suggest that HCC patients with absence of liver cirrhosis can benefit most from wide-margin LR.

Different from liver cirrhosis, which can be roughly judged by preoperative laboratory testing and intraoperative gross observation, MVI is a histopathological appearance that could be detected only with microscopy. It is almost implausible to make a decision on resection margin precisely based on the presence of MVI before hepatectomy. Therefore, the storm eye is whether it is enforceable to accurately predict the presence of MVI preoperatively. Scholars launched a great quantity of works about this topic. For instance, Banerjee et al. (39) used radiogenomic venous invasion, a contrast-enhanced CT biomarker, to predict MVI. The diagnostic accuracy, sensitivity, and specificity of it in predicting MVI were 89, 76, and 94%, respectively. Lee et al. (40) applied arterial peritumoral enhancement, non-smooth tumor margin, and peritumoral hypointensity on hepatobiliary phase based on gadoxetic acid–enhanced MRI to realize a >90% specificity of MVI prediction. Furthermore, artificial neural network and nomogram were also performed to develop reference to both imaging and laboratory test in order to make MVI prediction more diversified (41, 42). We also build a predicted model of MVI based on the patients in our study, which included ALP, AFP, PT, and tumor capsule. To a certain degree, this model could help in clinical screening of the population with high risk of MVI to adopt appropriate surgical strategies.

To our best knowledge, this study was the first to analyze the impact of surgical margin on the early HCC up to 5 cm and to suggest its correspondence with MVI and liver cirrhosis. However, the study still had several limitations. First, the study design was retrospective and therefore was subject to various inherent biases, which may have affected the reliability of some results. Second, the study involved designations of ≤2 mm as “narrow” and >2 mm as “wide” resection margins, whereas other studies used different values, which could present bias when comparing results across studies. The best surgical margin distance for various HCC patients still needs further exploration for more details and more individuals. Finally, our predictive model of MVI lacked external validation. Future studies will need to validate our findings in external population of both Eastern and Western patients.

## Conclusions

Among patients undergoing curative resection for solitary early HCC up to 5 cm, our study showed that a “wide-margin” (>2 mm) vs. “narrow-margin” (≤2 mm) surgical resection has a preponderant effect on both RFS and OS, especially for patients with presence of MVI and absence of liver cirrhosis. An MVI predictive model has also been proposed and recommended to be routinely used in the preoperative assessment to assist in the adoption of proper surgical procedures. Appropriate surgical margin should be assessed preoperatively based on both tumor factors and background liver factors.

## Data Availability Statement

The datasets generated for this study are available on request to the corresponding author.

## Ethics Statement

This study was approved by the Institutional Ethics Committee of the Eastern Hepatobiliary Surgery Hospital (NO. EHBHKY2015-02-001). The patients/participants provided their written informed consent to participate in this study. Written informed consent was obtained from the individual(s) for the publication of any potentially identifiable images or data included in this article.

## Author Contributions

Conception and critical revision: W-MC. Study design: W-MC and HW. Administrative support: M-CW. Data collection and acquisition: HW, HY, Y-WQ, and Z-YC. Data analysis: HW and HY. Manuscript preparation: HW and Y-WQ. Final approval of manuscript: all authors.

## Conflict of Interest

The authors declare that the research was conducted in the absence of any commercial or financial relationships that could be construed as a potential conflict of interest.

## Abbreviations

HCC: Hepatocellular carcinoma
LR: Liver resection
OS: Overall survival
TACE: Transcatheter arterial chemoembolization
MVI: Microvascular invasion
EHBH: Eastern Hepatobiliary Surgery Hospital
CT: Computed tomography
MRI: Magnetic resonance imaging
ES: Edmondson–Steiner
AFP: α-Fetoprotein
RFS: Recurrence-free survival
PSM: Propensity score matching
ALB: Albumin
GGT: γ-Glutamyl transpeptidase
ALP: Alkaline phosphatase
PLT: Platelets
PT: Prothrombin time
HBV DNA: Hepatitis B virus deoxyribonucleic acid
INR: International normalized ratio
AST: Aspartate aminotransferase.

## References

[1] Forner A Reig M Bruix J. Hepatocellular carcinoma. Lancet. (2018) 391:1301–14. 10.1016/S0140-6736(18)30010-2 29307467

[2] Nathan H Hyder O Mayo SC Hirose K Wolfgang CL Choti MA. Surgical therapy for early hepatocellular carcinoma in the modern era: a 10-year SEER-medicare analysis. Ann Surg. (2013) 258:1022–7. 10.1097/SLA.0b013e31827da749 23299519 PMC3994667

[3] Ng KKC Chok KSH Chan ACY Cheung TT Wong TCL Fung JYY. Randomized clinical trial of hepatic resection versus radiofrequency ablation for early-stage hepatocellular carcinoma. Br J Surg. (2017) 104:1775–84. 10.1002/bjs.10677 29091283

[4] Nathan H Schulick RD Choti MA Pawlik TM. Predictors of survival after resection of early hepatocellular carcinoma. Ann Surg. (2009) 249:799–805. 10.1097/SLA.0b013e3181a38eb5 19387322

[5] Huang G Lau WY Wang ZG Pan ZY Yuan SX Shen F. Antiviral therapy improves postoperative survival in patients with hepatocellular carcinoma: a randomized controlled trial. Ann Surg. (2015) 261:56–66. 10.1097/SLA.0000000000000858 25072444

[6] Wang H Du P-C Wu M-C Cong W-M. Postoperative adjuvant transarterial chemoembolization for multinodular hepatocellular carcinoma within the Barcelona Clinic Liver Cancer early stage and microvascular invasion. Hepatobil Surg Nutr. (2018) 7:418–28. 10.21037/hbsn.2018.09.05 30652086 PMC6295398

[7] Wei W Jian PE Li SH Guo ZX Zhang YF Ling YH. Adjuvant transcatheter arterial chemoembolization after curative resection for hepatocellular carcinoma patients with solitary tumor and microvascular invasion: a randomized clinical trial of efficacy and safety. Cancer Commun. (2018) 38:61. 10.1186/s40880-018-0331-y 30305149 PMC6235393

[8] Cong WM Wu MC. New insights into molecular diagnostic pathology of primary liver cancer: advances and challenges. Cancer Lett. (2015) 368:14–9. 10.1016/j.canlet.2015.07.043 26276723

[9] Feng LH Dong H Lau WY Yu H Zhu YY Zhao Y. Novel microvascular invasion-based prognostic nomograms to predict survival outcomes in patients after R0 resection for hepatocellular carcinoma. J Cancer Res Clin Oncol. (2016) 143:1–11. 10.1007/s00432-016-2286-1 27743138 PMC11819416

[10] Wang H Qian YW Wu MC Cong WM. Liver resection is justified in patients with BCLC intermediate stage hepatocellular carcinoma without microvascular invasion. J Gastrointest Surg. (2019). 10.1007/s11605-019-04251-8. [Epub ahead of print]. 31768830

[11] Wang H Wu MC Cong WM. Microvascular invasion predicts a poor prognosis of solitary hepatocellular carcinoma up to 2 cm based on propensity score matching analysis. Hepatol Res. (2019) 49:344–54. 10.1111/hepr.13241 30117236

[12] Jung DH Hwang S Lee YJ Kim KH Song GW Ahn CS. Small hepatocellular carcinoma with low tumor marker expression benefits more from anatomical resection than tumors with aggressive biology. Ann Surg. (2010) 269:1. 10.1097/SLA.0000000000002486 28837444

[13] Shimada S Kamiyama T Yokoo H Orimo T Wakayama K Einama T. Clinicopathological characteristics of hepatocellular carcinoma with microscopic portal venous invasion and the role of anatomical liver resection in these cases. World J Surg. (2017) 41:2087–94. 10.1007/s00268-017-3964-0 28271260

[14] Yang P Si A Yang J Cheng Z Wang K Li J. A wide-margin liver resection improves long-term outcomes for patients with HBV-related hepatocellular carcinoma with microvascular invasion. Surgery. (2018) 165:721–30. 10.1016/j.surg.2018.09.016 30554724

[15] Zhang E-L Liang B-Y Chen X-P Huang Z-Y. Severity of liver cirrhosis: a key role in the selection of surgical modality for Child-Pugh A hepatocellular carcinoma. World J Surg Oncol. (2015) 13:148. 10.1186/s12957-015-0567-9 25879526 PMC4427928

[16] Yang S-L Liu L-P Sun Y-F Yang X-R Fan J Ren J-W. Distinguished prognosis after hepatectomy of HBV-related hepatocellular carcinoma with or without cirrhosis: a long-term follow-up analysis. J Gastroenterol. (2016) 51:722–32. 10.1007/s00535-015-1146-0 26607653

[17] Beard RE Wang Y Khan S Marsh JW Tsung A Geller DA. Laparoscopic liver resection for hepatocellular carcinoma in early and advanced cirrhosis. HPB. (2018) 20:521–9. 10.1016/j.hpb.2017.11.011 29317157

[18] Berardi G Morise Z Sposito C Igarashi K Panetta V Simonelli I. Development of a nomogram to predict outcome after liver resection for hepatocellular carcinoma in Child-Pugh B cirrhosis. J Hepatol. (2020) 72:75–84. 10.1016/j.jhep.2019.08.032 31499131

[19] Sasaki K Shindoh J Margonis GA Nishioka Y Andreatos N Sekine A. Effect of background liver cirrhosis on outcomes of hepatectomy for hepatocellular carcinoma. JAMA Surg. (2017) 152:e165059. 10.1001/jamasurg.2016.5059 28052155

[20] Lee K-F Chong CCN Fong AKW Fung AKY Lok H-T Cheung Y-S. Pattern of disease recurrence and its implications for postoperative surveillance after curative hepatectomy for hepatocellular carcinoma: experience from a single center. Hepatobil Surg Nutr. (2018) 7:320–30. 10.21037/hbsn.2018.03.17 30498708 PMC6230848

[21] Cong WM Bu H Chen J Dong H Zhu YY Feng LH. Practice guidelines for the pathological diagnosis of primary liver cancer: 2015 update. World J Gastroenterol. (2016) 22:9279–87. 10.3748/wjg.v22.i42.9279 27895416 PMC5107692

[22] Sumie S Kuromatsu R Okuda K Ando E Takata A Fukushima N. Microvascular invasion in patients with hepatocellular carcinoma and its predictable clinicopathological factors. Ann Surg Oncol. (2008) 15:1375–82. 10.1245/s10434-008-9846-9 18324443

[23] Edmondson HA Steiner PE Primary carcinoma of the liver: a study of 100 cases among 48,900 necropsies. Cancer. (1954) 7:462–503. 10.1002/1097-0142(195405)7:3<462::aid-cncr2820070308>3.0.co;2-e 13160935

[24] Pang YY The Brisbane 2000 terminology of liver anatomy and resections. HPB. (2002) 2:333–39. 10.1080/136518202760378489 PMC2020531 18332933

[25] Oguro S Yoshimoto J Imamura H Ishizaki Y Kawasaki S. Clinical significance of macroscopic no-margin hepatectomy for hepatocellular carcinoma. HPB. (2018) 20:872–80. 10.1016/j.hpb.2018.03.012 29699859

[26] Field WBS Rostas JW Philps P Scoggins CR McMasters KM Martin RCG II. Wide versus narrow margins after partial hepatectomy for hepatocellular carcinoma: balancing recurrence risk and liver function. Am J Surg. (2017) 214:273–7. 10.1016/j.amjsurg.2017.06.002 28615138

[27] Yu H Wang H Xu HR Zhang YC Yu XB Wu MC. Overexpression of MTHFD1 in hepatocellular carcinoma predicts poorer survival and recurrence. Fut Oncol. (2019) 15:1771–80. 10.2217/fon-2018-0606 30997850

[28] Rubin DB Thomas N. Matching using estimated propensity scores: relating theory to practice. Biometrics. (1996) 52:249–64. 10.2307/2533160 8934595

[29] Llovet JM Bru C Bruix J. Prognosis of hepatocellular carcinoma: the BCLC staging classification. Semin Liver Dis. (1999) 19:329–38. 10.1055/s-2007-1007122 10518312

[30] Poon RT Fan ST Ng IO Wong J. Significance of resection margin in hepatectomy for hepatocellular carcinoma: a critical reappraisal. Ann Surg. (2000) 231:544–51. 10.1097/00000658-200004000-00014 10749616 PMC1421031

[31] Shi M Guo RP Lin XJ Zhang YQ Chen MS Zhang CQ. Partial hepatectomy with wide versus narrow resection margin for solitary hepatocellular carcinoma: a prospective randomized trial. Ann Surg. (2007) 245:36–43. 10.1097/01.sla.0000231758.07868.71 17197963 PMC1867934

[32] Pan YX Long Q Yi MJ Chen JB Chen JC Zhang YJ. Radiofrequency ablation versus laparoscopic hepatectomy for hepatocellular carcinoma: a real world single center study. Eur J Surg Oncol. (2019) 46(Pt. A):548–59. 10.1016/j.ejso.2019.10.026 31677940

[33] Zhao W-J Zhu G-Q Wu Y-M Wang W-W Bai B-L. Comparative effectiveness of radiofrequency ablation, surgical resection and transplantation for early hepatocellular carcinoma by Cancer Risk Groups: results of propensity score-weighted analysis. Onco Targets Ther. (2019) 12:10389–400. 10.2147/OTT.S224809 31819521 PMC6890195

[34] Park JH Dong HK Kim SH Kim MY Baik SK Hong IS. The clinical implications of liver resection margin size in patients with hepatocellular carcinoma in terms of positron emission tomography positivity. World J Surg. (2017) 42–1–9. 10.1007/s00268-017-4275-1 29026966

[35] Wbs F Rostas JW Philps P Scoggins CR Mcmasters KM Wide versus narrow margins after partial hepatectomy for hepatocellular carcinoma: balancing recurrence risk and liver function. Am J Surg. (2017) 18:e229 10.1016/j.hpb.2016.02.572 28615138

[36] Lee W Han HS Ahn S Yoon YS Cho JY Choi Y. Correlation between resection margin and disease recurrence with a restricted cubic spline model in patients with resected hepatocellular carcinoma. Dig Surg. (2018) 35:520–31. 10.1159/000485805 29342456

[37] Lafaro K Grandhi MS Herman JM Pawlik TM. The importance of surgical margins in primary malignancies of the liver. J Surg Oncol. (2016) 113:296–303. 10.1002/jso.24123 26659586

[38] Lee JI Lee HW Kim SU Ahn SH Lee KS. Follow-up liver stiffness measurements after liver resection influence oncologic outcomes of hepatitis-B-associated hepatocellular carcinoma with liver cirrhosis. Cancers. (2019) 11:425. 10.3390/cancers11030425 30934621 PMC6468874

[39] Banerjee S Wang DS Kim HJ Sirlin CB Chan MG Korn RL. A computed tomography radiogenomic biomarker predicts microvascular invasion and clinical outcomes in hepatocellular carcinoma. Hepatology. (2015) 62:792–800. 10.1002/hep.27877 25930992 PMC4654334

[40] Lee S Kim SH Lee JE Sinn DH Park CK. Preoperative gadoxetic acid-enhanced MRI for predicting microvascular invasion in patients with single hepatocellular carcinoma. J Hepatol. (2017) 67:526–34. 10.1016/j.jhep.2017.04.024 28483680

[41] Alessandro C Fabio P Antonia D'Errico G Matteo R Matteo C Matteo Z Preoperative prediction of hepatocellular carcinoma tumour grade and micro-vascular invasion by means of artificial neural network: a pilot study. J Hepatol. (2010) 52:880–8. 10.1016/j.jhep.2009.12.037 20409605

[42] Lei Z Li J Wu D Xia Y Wang Q Si A. Nomogram for preoperative estimation of microvascular invasion risk in hepatitis B virus-related hepatocellular carcinoma within the Milan criteria. JAMA Surg. (2016) 151:356–63. 10.1001/jamasurg.2015.4257 26579636

