# Supplementary material for: Impact of Surgical Margin on the Prognosis of Early Hepatocellular Carcinoma (≤5 cm): A Propensity Score Matching Analysis
Source: Front Med (Lausanne). 2020 May 7;7:139. doi: 10.3389/fmed.2020.00139 (PMC7232563; doi:10.3389/fmed.2020.00139)
Supplement: Supplementary file 1 [file Data_Sheet_1.docx]

| Supplementary Table 1 Results of X-tile. | | | | | |
| --- | --- | --- | --- | --- | --- |
| Surgical margin distance | RFS | | OS | | Number of "wide-" and "narrow-" margin group |
|  | Chi-square value | P value | Chi-square value | P value |  |
| 1 mm | 10.8406 | 0.0259 | 16.3522 | 0.0019 | 568:336 |
| 2 mm | 37.9838 | <0.0001 | 18.2927 | 0.0008 | 464:440 |
| 3 mm | 23.6210 | <0.0001 | 16.0142 | 0.0022 | 385:519 |
| 4 mm | 13.0925 | 0.0091 | 8.0599 | 0.0860 | 317:587 |
| 5 mm | 7.5018 | 0.1116 | 6.2889 | 0.1858 | 296:608 |
| 6 mm | 3.6525 | 0.5174 | 6.5240 | 0.1709 | 247:657 |
| 7 mm | 2.8920 | 0.6786 | 6.6660 | 0.1571 | 220:684 |
| 8 mm | 2.1994 | 1.0000 | 4.5520 | 0.3696 | 197:707 |
| 9 mm | 1.9767 | 1.0000 | 4.5244 | 0.3841 | 186:718 |
| 1 cm | 2.5974 | 1.0000 | 5.1956 | 0.2918 | 184:720 |
| 1.5 cm | 0.3074 | 1.0000 | 0.0092 | 1.0000 | 80:824 |
| 2 cm | 0.1284 | 1.0000 | 0.0051 | 1.0000 | 50:854 |

| Supplementary Table 2 Predictive factors of microvascular invasion based on the PSM group. | | | | |
| --- | --- | --- | --- | --- |
| Variables | Univariable | | Multivariable | |
|  | OR (95%CI) | P value | OR (95%CI) | P value |
| Sex, male | 0.698 (0.458, 1.063) | 0.094 |  |  |
| Age, years | 0.984 (0.968, 0.999) | 0.042 |  |  |
| TBIL, μmol/L | 1.017 (0.989, 1.046) | 0.232 |  |  |
| TP, g/L | 1.002 (0.975, 1.030) | 0.875 |  |  |
| ALB, g/L | 0.986 (0.946, 1.028) | 0.505 |  |  |
| ALT, U/L | 1.000 (0.994, 1.005) | 0.871 |  |  |
| AST, U/L | 1.005 (0.997, 1.013) | 0.252 |  |  |
| GGT, U/L | 1.001 (0.999, 1.003) | 0.410 |  |  |
| ALP, U/L | 1.011 (1.004, 1.018) | 0.002 | 1.011 (1.004, 1.018) | 0.003 |
| AFP, ng/mL | 1.001 (1.000, 1.001) | <0.001 | 1.001 (1.000, 1.001) | <0.001 |
| CA199, ng/mL | 1.001 (0.994, 1.008) | 0.715 |  |  |
| WBC, ×10^9^/L | 0.941 (0.855, 1.036) | 0.217 |  |  |
| RBC, ×10^9^/L | 0.793 (0.582, 1.081) | 0.143 |  |  |
| PLT, ×10^9^/L | 0.997 (0.995, 1.000) | 0.074 |  |  |
| INR | 10.416 (1.551, 69.965) | 0.016 |  |  |
| PT, s | 1.208 (1.032, 1.414) | 0.019 | 1.193 (1.015, 1.403) | 0.032 |
| HBsAg, positive | 1.097 (0.638, 1.887) | 0.738 |  |  |
| HBsAb, positive | 1.060 (0.671, 1.672) | 0.804 |  |  |
| HBeAg, positive | 0.744 (0.528, 1.049) | 0.092 |  |  |
| HBeAb, positive | 1.400 (0.967, 2.026) | 0.075 |  |  |
| HBcAb, positive | 1.320 (0.240, 7.258) | 0.750 |  |  |
| HBV DNA load, >10^3^IU/mL | 0.892 (0.654, 1.215) | 0.467 |  |  |
| Child Pugh, B | 2.041 (0.453, 9.192) | 0.353 |  |  |
| Hepatectomy, anatomical | 1.073 (0.704, 1.634) | 0.743 |  |  |
| Transfusion, yes | 1.121 (0.665, 1.889) | 0.669 |  |  |
| Pringle maneuver, yes | 0.775 (0.496, 1.210) | 0.262 |  |  |
| Diameter, cm | 1.135 (0.974, 1.322) | 0.105 |  |  |
| Cirrhosis, yes | 1.838 (1.321, 2.558) | <0.001 |  |  |
| Capsule, no | 1.666 (1.064, 2.608) | 0.026 | 1.736 (1.094, 2.756) | 0.019 |
| ES grade, Ⅲ-Ⅳ | 3.868 (2.656, 5.633) | <0.001 |  |  |
| Surgical margin, narrow | 0.928 (0.681, 1.265) | 0.636 |  |  |
| PSM propensity score matching, TBIL total bilirubin, TP total protein, ALB albumin, ALT alanine transaminase, AST aspartate aminotransferase, GGT γ-glutamyl transpeptidase, ALP alkaline phosphatase, AFP alpha fetal protein, CA199 carbohydrate antigen 19-9, WBC white blood cells, RBC red blood cells, PLT platelets, INR international normalized ratio, PT prothrombin time, HBsAg Hepatitis B surface antigen, HBsAb Hepatitis B surface antibody, HBeAg Hepatitis B e antigen, HBeAb Hepatitis B e antibody, HBcAb Hepatitis B c antibody, HBV DNA Hepatitis B virus deoxyribonucleic acid, MVI microvascular invasion, ES Edmondson-Steiner. | | | | |

| Supplementary Table 3 Pattern of recurrence for the whole patients. | | | |
| --- | --- | --- | --- |
| Characteristics | Wide margin (N=186) | Narrow margin (N=260) | P value |
| Recurrence site |  |  | 0.612 |
| Intrahepatic | 173 (93.0%) | 247 (95.0%) |  |
| Extrahepatic | 10 (5.4%) | 9 (3.5%) |  |
| Intrahepatic+Extrahepatic | 3 (1.6%) | 4 (1.5%) |  |
| Intrahepatic tumor number |  |  | 0.003 |
| Single nodule | 138 (75.4%) | 159 (62.1%) |  |
| Multiple nodules | 45 (24.6%) | 97 (37.9%) |  |
| Macrovascular invasion |  |  | 0.164 |
| Yes | 5 (2.7%) | 14 (5.4%) |  |
| No | 181 (97.3%) | 246 (94.6%) |  |
| Treatment method |  |  | 0.230 |
| Re-resection | 32 (17.2%) | 37 (14.2%) |  |
| Ablation | 46 (24.7%) | 48 (18.5%) |  |
| TACE | 93 (50.0%) | 154 (59.2%) |  |
| Others | 15 (8.1%) | 21 (8.1%) |  |
| TACE transcatheter arterial chemoembolization. | | | |


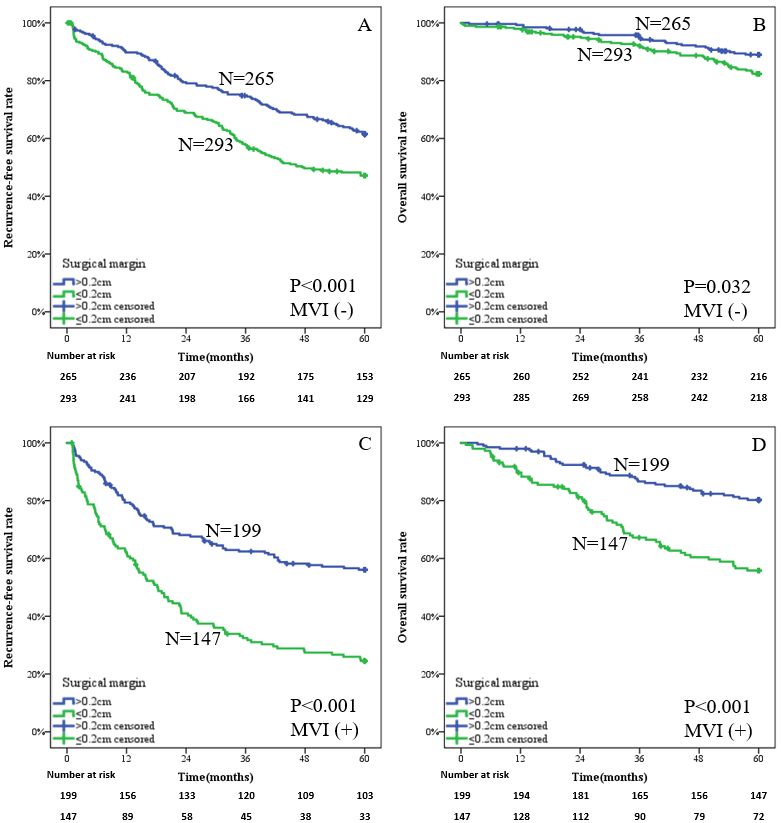


Supplementary Figure 1. Subgroup analysis of a wide-margin versus a narrow-margin liver resection for the whole patients. (A) recurrence-free survival in the patients without microvascular invasion, (B) overall survival in the patients without microvascular invasion, (C) recurrence-free survival in the patients with microvascular invasion, (D) overall survival in the patients with microvascular invasion.


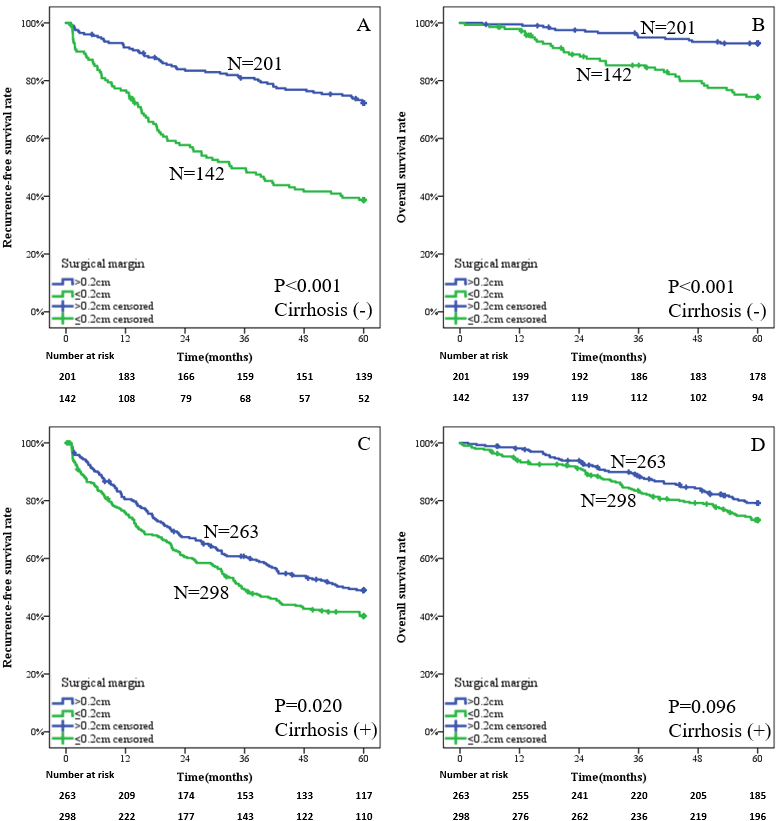


Supplementary Figure 2. Subgroup analysis of a wide-margin versus a narrow-margin liver resection for the whole patients. (A) recurrence-free survival in the patients without liver cirrhosis, (B) overall survival in the patients without liver cirrhosis, (C) recurrence-free survival in the patients with liver cirrhosis, (D) overall survival in the patients with liver cirrhosis.
